# Coriolis and centrifugal forces drive haltere deformations and influence spike timing

**DOI:** 10.1098/rsif.2019.0035

**Published:** 2019-04-24

**Authors:** T. L. Mohren, T. L. Daniel, A. L. Eberle, P. G. Reinhall, J. L. Fox

**Affiliations:** 1 Department of Mechanical Engineering, University of Washington, Seattle, WA, USA; 2 Department of Biology, University of Washington, Seattle, WA, USA; 3 Department of Biology, Case Western Reserve University, Cleveland, OH, USA

**Keywords:** haltere, gyroscopic sensing, finite-element analysis, neural encoding

## Abstract

The halteres of flies are mechanosensory organs that serve a crucial role in the control of agile flight, providing sensory input for rapid course corrections to perturbations. Derived from hind wings, halteres are actively flapped and are thus subject to a variety of inertial forces as the fly undergoes complex flight trajectories. Previous analyses of halteres modelled them as a point mass, showing that Coriolis forces lead to subtle deflections orthogonal to the plane of flapping. By design, these models could not consider the effects of force gradients associated with a mass distribution, nor could they reveal three-dimensional spatio-temporal patterns of strain that result from those forces. In addition, diversity in the geometry of halteres, such as shape and asymmetries, could not be simply modelled with a point mass on a massless rod. To study the effects of mass distributions and asymmetries, we examine the haltere subject to both flapping and body rotations using three-dimensional finite-element simulations. We focus on a set of simplified geometries, in which we vary the stalk and bulb shape. We find that haltere mass distribution gives rise to two unreported deformation modes: (i) halteres twist with a magnitude that strongly depends on stalk and bulb geometry and (ii) halteres with an asymmetric mass distribution experience out-of-plane bending due to centrifugal forces, independent of body rotation. Since local strains at the base of the haltere drive deformations of mechanosensory neurons, we combined measured neural encoding mechanisms with our structural analyses to predict the spatial and temporal patterns of neural activity. This activity depends on both the flapping and rotation dynamics, and we show how the timing of neural activity is a viable mechanism for rotation-rate encoding. Our results provide new insights in haltere dynamics and show the viability for timing-based encoding of fly body rotations by halteres.

## 1. Introduction

Animals control movement via the integration of inputs from multiple sensory modalities. In insect flight control, sensory inputs are largely dominated by both visual and mechanosensory systems. In the extremely rapid dynamics associated with insect flight, feedback control via visual input is often too slow to provide adjustments to the flight path in response to perturbations [1–3], leading to pitch instabilities. To compensate for relatively slower visual input, rapid feedback from mechanosensory structures often serves a crucial role in flight control [4].

In dipteran insects (the true flies), hindwings have evolved into specialized mechanosensory structures called halteres. These organs provide exceedingly rapid feedback information about the animal’s body dynamics [5]. As halteres flap and the body rotates, Coriolis forces drive subtle deformations of the halteres that are perpendicular to the plane of flapping. Coriolis forces are directly proportional to both the flapping frequency and the body rotation rate [6–8]. The dynamics associated with Coriolis-induced motions have been widely studied over the greater part of the last century, with a consensus view that gyroscopic sensing capabilities follow from the detection of small out-of-plane bending [6–8] (for the mathematical basis see the electronic supplementary material). This out-of-plane deflection is transformed into neural signals via mechanical sensory structures, called campaniform sensilla, located in dense fields at the haltere’s base. These sensilla provide rapid flight feedback via fast electrotonic synapses onto the flight motor neurons [9]. Sensory information from the haltere nerve is also important in gaze stabilization [10,11]. While there is significant behavioural and electrophysiological evidence for sensory roles of halteres [6,12,13], the tiny, rotation-induced bending deformations have not been observed experimentally. In addition, it has been historically thought that detection of such small lateral deflections would require strong directional sensitivity of the campaniform sensilla. While some animals, such as spiders, are known to employ mechanosensors, called slit sensilla, which possess high directional sensitivity [14–16], whether the directionality of strain is encoded by campaniform sensilla on halteres remains unclear.

**Figure 1.**
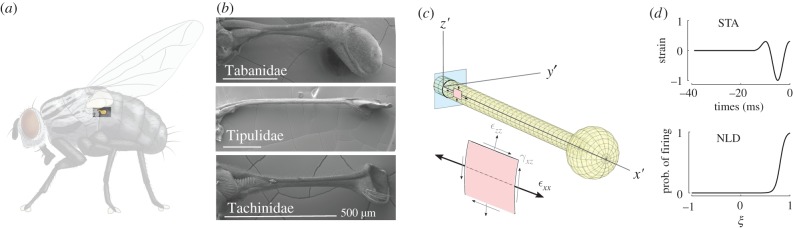
Insects belonging to the order of the true flies (Diptera) possess halteres—dumbbell-shaped mechanosensory organs associated with inertial sensing (*a*). Three scanning electron microscope images show different haltere geometries associated with specimens from three different genera (*b*; courtesy of Sweta Agrawal). The geometry of our basic finite-element mode (*c*) with normal and shear strain components near the base of the haltere. (*d*) The normalized spike-triggered average (STA) and nonlinear decision function (NLD) derived from the encoding properties of cranefly haltere neurons. The parameter *ξ* is the normalized projection of the STA onto the stimulus from a white noise analysis. Both the STA and NLD are derived from Fox *et al.* [13]. (Online version in colour.)

Prior analytic studies of halteres have modelled them as a single point mass at the end of a massless rod, allowing only deformations that result from both Coriolis forces and in-plane inertial bending [6–8]. By contrast, more recent models of insect wings suggest that Coriolis forces on distributed masses lead to torsional deformations which had not been previously considered [17]. Indeed, stabilizing reflexes have been observed in moths in response to magnetically induced wing twist [18]. It is thus possible that the distribution of mass in halteres could similarly yield torsional dynamics, even if it is symmetrically distributed with respect to the stalk. Moreover, asymmetries abound, with impressive geometric variation of halteres across a wide range of taxa [19] (see example in figure 1*b*). In addition, asymmetric mass distributions of the bulb could potentially lead to relatively large out-of-plane deformations due to centrifugal forces, an additional component of haltere dynamics that had not been examined previously. Thus two modes of deformation—torsion and centrifugally generated bending—remain unexplored. Here, we ask four key questions. What are the three-dimensional deformations, stresses and strains in halteres when undergoing concurrent flapping and rotation? What are the dominant forces leading to those deformations? How do the structural features of halteres influence the relative magnitudes of emergent deformations? And how do such deformations ultimately lead to patterns of neural activation? Answers to these questions will guide a deeper understanding of haltere design and function, as well as the roles of sensory information and spike timing [20] in insect flight control. Moreover, the mechanisms by which complex body dynamics are transformed into neural signals via the deformations of halteres can inspire novel design for synthetic gyroscopic sensors.

In contrast to prior studies of halteres that used point mass simulations, we address these questions using three-dimensional finite-element models to examine spatial and temporal patterns of the deformations and strains associated with haltere dynamics. We combine strain data from the finite-element models with neural encoding properties obtained from electrophysiological recordings to develop predictive models of spatial and temporal patterns of neural signals from campaniform sensilla at the haltere base. We find that both centrifugal forces and Coriolis-induced torsion contribute to the strain patterns at the base of the haltere, and that these forces can influence the timing of neural activation. Moreover, the geometry of halteres influences the relative contributions of these forces in the mechanism of motion encoding. Taken together, our results suggest a new view of the mechanics and neurobiology of rotation sensing in flying insects.

## 2. Material and methods

Our model of the haltere geometry and kinematics in the structural simulations was inspired by the cranefly haltere from the family Tipulidae. We assumed homogeneous material properties and a cylindrical stalk geometry to establish a mechanistic understanding of the deformation modes.

### 2.1. Geometry and kinematics

Our base model consisted of a hollow stalk of circular cross-section with an outer radius of 150 μm and an inner radius of 50 μm. The end of the stalk supported a bulb with the centre of mass at a distance of 5 × 10^3^ μm from the base of the haltere. The bulb had a radius of 500 μm.

We assume uniform material properties, and use a Young's modulus of *E* = 1.5 GPa, as obtained from nanoindentation on halteres [21]. We set both the stalk and bulb density to *ρ* = 1200 kg m^−3^ to be consistent with previous modelling [21] and with prior estimates of cuticle stiffness [22]. Furthermore, we assume a Poisson ratio of *ν* = 0.33. We note that Young’s modulus of scerotized cuticle can range from 1 to 20 GPa, and its density falls between 1 and 1.3 kg m^−3^ [23]. We modelled the bulb as a rigid element, as the moment due to gyroscopic accelerations is smallest at the tip and results in a small contribution to the total haltere deformation.

**Figure 2.**
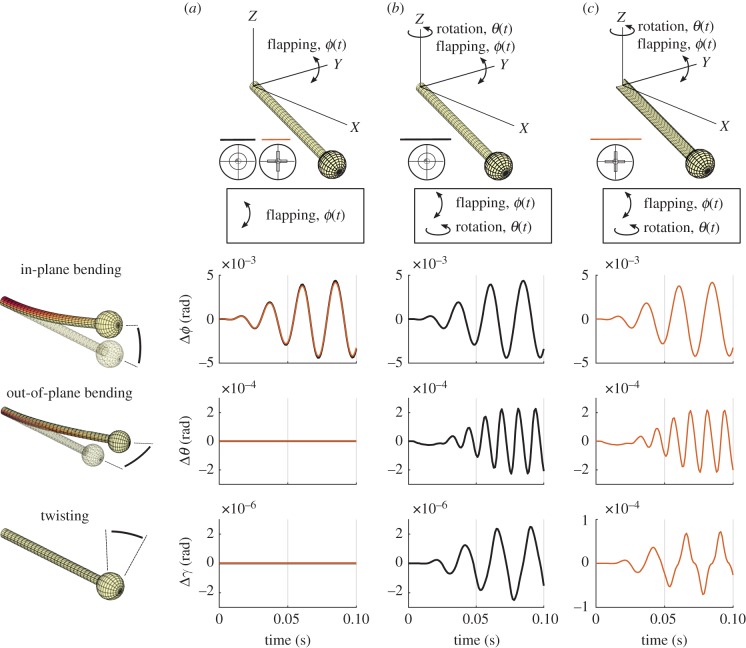
Three deformation modes are shown for two different stalk geometries. (*a*) The graphs represent the deformations that result from pure flapping motions with two stalk geometries: circular cross-section (CS, black) and a plus-sign-shaped cross-section (PS, red). The deformations that occur for the CS cross-section (*b*) and the PS cross-section (*c*) experiencing both flapping and rotation show the importance of torsional stiffness relative to bending stiffness. Note the different axis magnitudes for the CS-shaped stalk demonstrating the importance of the polar moment of area relative to the second moment of area. (Online version in colour.)

**Figure 3.**
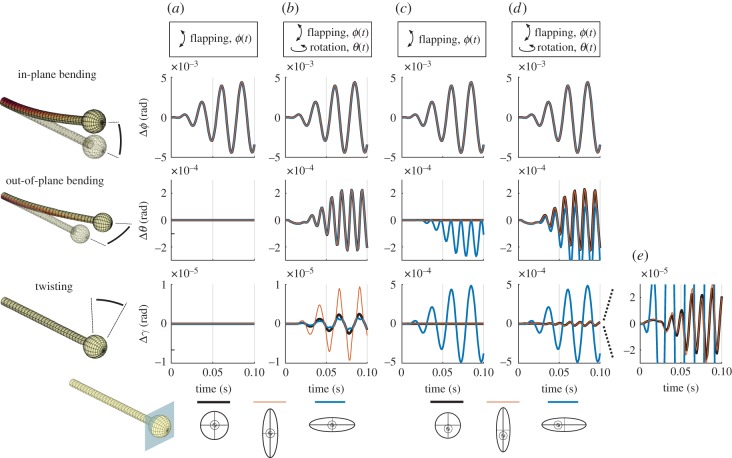
Three deformation modes are shown for different bulb geometries (*a* and *b* are symmetrically mounted, *c* and *d* are asymmetric). In (*a*) both symmetrically mounted ellipsoidal and spherical bulbs have identical deformations when experiencing only flapping motions (all three therefore have the same line colour). (*b*) The predicted deformations for the three symmetrically mounted bulbs with identical deformations for in-plane and out-of-plane bending experiencing flapping and rotation. Torsional motions are greatest for the vertically oriented bulb. The predicted deformations for asymmetrically mounted bulbs experiencing only flapping (*c*) and for both flapping and rotations (*d*) show the influence of both Coriolis and centrifugal forces. (*e*) An expansion of the twisting deformations in (*d*). Note the different axis magnitudes. (Online version in colour.)

To investigate the dependence of haltere deformation on haltere shape, we modelled two different stalk cross-sections and three bulb geometries. We compared the spherical bulb with elliptical bulbs with a major axis length of 1000 μm and two minor axis lengths of approximately 353 μm, maintaining the same internal volume and centre-of-mass distance from the base of the haltere (figure 3). To investigate the effect of asymmetry, we also offset the bulb centre of mass in either the *y*′ or *z*′ direction by 150 μm. To examine the role of torsional and bending stiffness of the stalk, we modelled both the circular-shaped cross-section (CS) as described previously, as well as a plus-sign-shaped cross-section (PS) consisting of two orthogonal rectangles with a height of 481 μm and a thickness of 40.8 μm, corresponding to an aspect ratio of 12. These two cross-sectional shapes were scaled to have an identical second moment of area *I*, but a vastly different torsional moment of inertia *J* (by an approximate factor of 38; figure 2; derivation in the electronic supplementary material).

We fixed the base of the haltere model to a rigid base plate that we rotated in the *Y*- and *Z*-axis. We prescribed the flapping motion as the haltere stroke angle about the *Y*-axis,

2.1 $$\phi (t)=A_{f}\sin(2\pi f_{\phi }t)\;\left[\text{rad}\right],$$

where $f_{\phi }=40$ Hz and amplitude *A*_*f*_ = *π*/2 rad, values that approximate observations of cranefly halteres [12,24]. Interestingly, the haltere flapping frequency is mechanically coupled to wingbeat frequency [25].

We then specified one of two different rotational velocities to the haltere frame around the *Z*-axis,

2.2 $$\begin{array}{r} {\dot{\theta }}_{\;\mathrm{Flapping}\;\mathrm{only}}=0\;[{\text{rad s}}^{-1}]\; \end{array}$$

and

2.3 $${\dot{\theta }}_{\mathrm{With}\;\mathrm{rotation}}=10\;[{\text{rad s}}^{-1}].$$

We chose a conservative rotation rate of 10 rad s^−1^ since yaw rates between 14 and 28 rad s^−1^ are commonly observed in flying insects [26]. Both the flapping and rotation were ramped up using a sigmoidal function during the start-up phase.

We used a radially symmetric mesh for the stalk using linear hexahedral elements (see also figure 1*c*). We specified both the inner and outer circumference of the stalk to have 16 equally spaced nodes. We prescribed five nodes at equal radial distances, resulting in four by four elements per stalk quadrant. We used 31 elements along the length of the stalk. The bulb is composed of four elements per quadrant in both curved directions. We added Rayleigh damping of *α*_*k*_ = 1 × 10^−5^ and *α*_*m*_ = 0 to ensure the stability of our solution. The selection of damping values do not change the results within two orders of magnitude of the value we selected (see the electronic supplementary material). However, large damping values can counter-intuitively lead to instabilities. Such damping-induced dynamics may relate to a phenomenon known as damping-induced rotational instabilities [27]. Our simulations were implemented in COMSOL 5.0.

### 2.2. Deformation and strain

We took the location of the two nodes at the extreme sides of the haltere bulb to calculate the deformation angle from the deformations *δ* in the haltere’s local *x*′*y*′*z*′-reference frame with in-plane bending, Δ*ϕ* = tan (*δ*_*z*_/*x*_*d*_), and out-of-plane bending, Δ*θ* = tan(*δ*_*y*_/*x*_*d*_), where *x*_*d*_ is the distance from the stalk base to the bulb centre of mass. We used the two side points to calculate the twist angle Δ*γ* = tan (*δ*_*z*_/*y*_*d*_), with *δ*_*z*_ the distance between the two points along the *z*′-axis, and *y*_*d*_ the distance between the two points along the *y*′-axis. In this study, we used the normal strain in the *x*′-direction of the local frame (*ε*_*xx*_), as it is the dominant strain signal. We exported this strain from the finite-element software, but we note that strain relates to the local curvature according to the Euler–Bernoulli beam theory in thin beams experiencing small deflections [28],

2.4 $$\epsilon_{xx}=-z\frac{{\text{d}}^{2}w}{\text{d}x^{2}},$$

with *z* the distance with respect to the neutral axis of the beam and *w* the deflection in the *z*′-axis.

### 2.3. Neural encoding

To predict spiking from our simulations, we used normal strain in the spanwise direction of the haltere (*ε*_*xx*_), at the locations along the outer circumference of the haltere and two mesh nodes outward from the base (300 μm distally). We focus here on a single example geometry to illustrate the transformation of spatio-temporal patterns of strain to patterns of neural activation. This strain was passed through a two-step spike prediction method, using the neural encoding properties obtained from electrophysiological recordings from cranefly haltere neurons following methods outlined in [12,13,29,30]. These recordings revealed the temporal patterns of mechanical strain stimuli that would most likely lead to an action potential from haltere neurons. The average temporal pattern that elicits a neuron to fire, the spike-triggered average (STA), was derived from averaging across the ensemble of stimulus histories preceeding the spikes of that neuron. Given the STA and the distribution of stimuli that generate action potentials, we used Bayes’ theorem to identify the probability of action potential generation for a stimulus. This probability of firing followed a sigmoidal curve, often referred to as the nonlinear decision function (NLD). With the STA and NLD, we then determined the probability of action potentials occurring over time for any given temporal strain signal. We used STA and NLD estimates from a white noise motion stimulus provided to the tip of the haltere [13]. We used methods outlined in [13] to scale the displacement stimulus to strain at the base of the haltere. We assume that a spike occurs at the peak of the probability of firing, but only if this peak probability of firing exceeded a threshold value of 0.9. The transformation from strain to firing rate and spiking is shown in figure 1*d*.

The simulation data from the computational models are available at Zenodo (https://doi.org/10.5281/zenodo.2542944). We used Matlab [31] to analyse the data. Our code to analyse the finite-element results and to implement the neural encoding can be found at https://github.com/tlmohren/Haltere-code.

## 3. Results

Our results are consistent with those of earlier studies that indicated significant in-plane bending and a subtle out-of-plane bending induced by the Coriolis force acting on a flapping and rotating haltere [6–8] (figure 2). For our flapping haltere, not subject to Coriolis forces, the in-plane bending attains a peak-to-peak amplitude of approximately 0.01 rad. That amplitude is about 25 times greater than the out-of-plane amplitude when the haltere is subject to Coriolis forces resulting from the orthogonal body rotations we imposed in the model. Moreover, the frequency of the out-of-plane bending is twice that of the flapping frequency, a result consistent with all prior studies of halteres [6–8]. Interestingly, a haltere with a spherical bulb and a cylindrical shaft also experiences torsional deformation (twisting) which is much smaller in amplitude than either the in-plane or out-of-plane bending (figure 2*b*, blue lines). That torsional deformation has a dominant frequency that is at the same frequency as the flapping motions.

*Stalk geometry*. Circular cross-sections (CS) represent structures that have the highest resistance to torsional deformations: their polar moment of area *J* is maximum for a given amount of material. Haltere stalks are not perfectly circular. Instead, they have a groove running along the stalk that reduces their polar moment of area [19]. To explore the consequences of different beam cross-sections, we created the plus-shaped cross-section (PS) that has the same second moment *I* of area as a cylinder, but a significantly lower *J*. This PS cross-section has an identical out-of-plane displacement as we found for a circular cross-section (figure 2*c*, middle). However, the torsional deformation of the PS cross-section is much greater than that for the CS cross-section and of same order of magnitude as the out-of-plane deformation (figure 2*c*, bottom).

*Haltere asymmetries*. Because haltere deformations depend quite strongly on the distribution of mass, we explored the consequences of two geometric scenarios. In one case, we asked how deformations depend on the distribution of mass for bulbs symmetrically mounted to a shaft with the same CS cross-section. In this scenario, we compared the deformations of halteres with ellipsoidal bulbs with that of a spherical bulb, all with the same total mass. All three bulb shapes led to identical out-of-plane bending deformations when subject to both flapping and rotation. None demonstrated out-of-plane or torsional deformations for flapping only (figure 3*a*). Interestingly, mass distributions that are symmetric with respect to the shaft and oriented in the plane of the body rotation and parallel to the plane of flapping (figure 3*b*, vertical bulb, red lines) experience much greater twisting than that of either a spherical bulb (black line) or an ellipsoidal bulb oriented orthogonally to the plane of flapping (horizontal bulb, blue line).

Even for a haltere with an asymmetric bulb, solely undergoing flapping excitation, an additional deformation mode arises (figure 3*c*). First, for a horizontal ellipsoid bulb, a centrifugal force (see electronic supplementary material for the equations) leads to out-of-plane bending motions that are of the same magnitude as those seen for halteres experiencing Coriolis forces (figure 3*c*, middle, blue line). In this instance, centrifugal forces yield deformations at twice the frequency of the flapping motion, which is the same frequency as occurs with Coriolis forces. Moreover, there is a net offset to the bending angle of the haltere resulting from a lateral torque due to centrifugal forces. The vertical ellipsoidal bulb which is asymmetrically mounted on the shaft (figure 3*c*, middle, red line) also experiences centrifugal forces, but these are manifest as subtle changes in the in-plane bending (the difference for in-plane bending is only 1 × 10^−4^ rad).

When rotation is added, both asymmetrically mounted ellipsoidal bulbs experience large out-of-plane bending at twice the flapping frequency due to Coriolis forces (figure 3*d*, middle). The vertically oriented ellipsoidal bulb (red line) and spherical bulb (black line) have similar out-of-plane bending motions. However, the horizontal ellipsoidal bulb experiences significant twisting motions, driven largely by the high-amplitude flapping motions (figure 3*c*,*d*, bottom), with a modest Coriolis-induced torsion that is also apparent for vertical ellipsoidal bulb.

**Figure 4.**
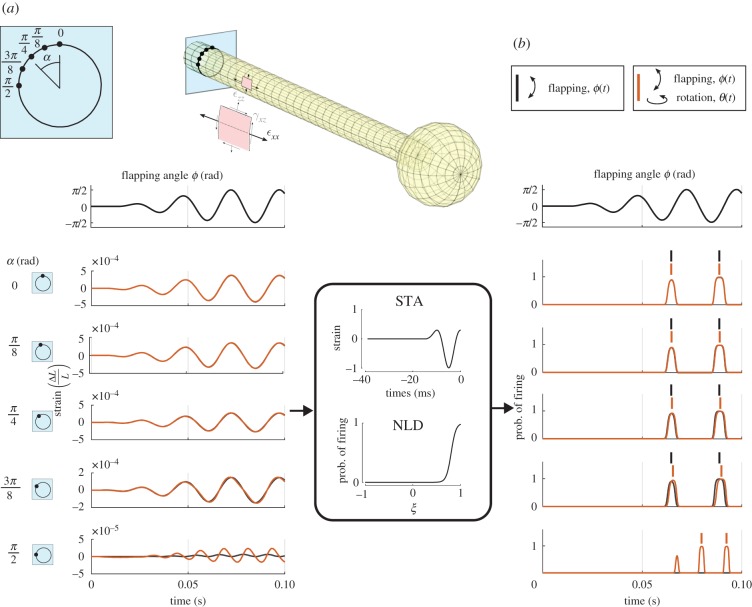
(*a*,*b*) The strain along the first quarter of the haltere circumference for flapping and flapping with rotation is converted into probability of firing through the neural encoding properties from haltere campaniforms, as described in [13]. The spike timing on the top of the haltere does not change due to rotation, but for larger *α* the spikes have an increasing timing difference. Finally, at the sides of the haltere, there are no more spikes without rotation. We use strain in the longitudinal direction (indicated with *ε*_*xx*_ on the shell element). (Online version in colour.)

*Spike timing along circumference.* The temporal pattern of normal strain at five locations around the circumference of the shaft was processed with a neural filter to predict the probability of neuronal firing (spiking) as a function of angular position around the base of the haltere (figure 4) subject to both flapping and rotation. The normal strain at the top is dominated by in-plane bending, whereas the normal strain at the side is dominated by the Coriolis force. There is also a modest normal strain present on the side (figure 4*a*, black line) and everywhere along the haltere that arises from centrifugal forces tensioning the haltere shaft because of the flapping motion. The difference in spike times with Coriolis forces present increases towards the lateral positions of the haltere base. At an angle *α* = *π*/2 rad, neurons only spike in the presence of rotations and do so at twice the flapping frequency.

**Figure 5.**
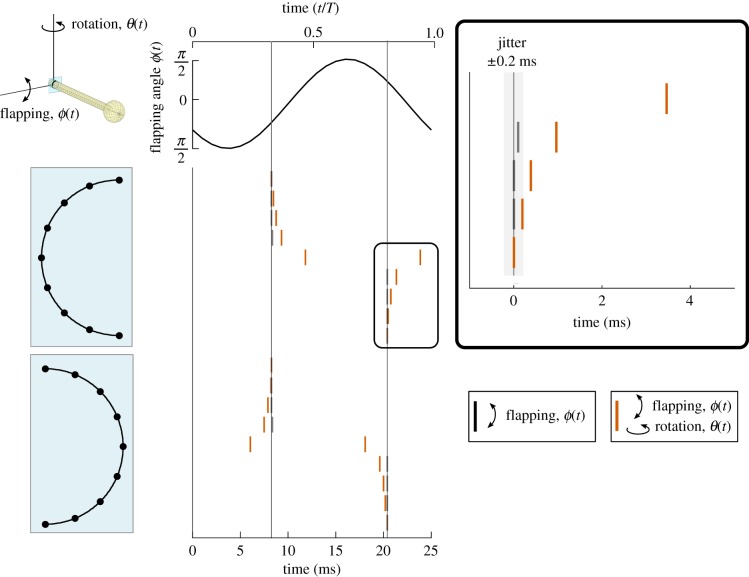
We use the spike prediction method to predict spiking along the circumference of the haltere for flapping (black stripes), and flapping with rotation (red stripes). At the side extremes, no spikes occur unless the haltere rotates, and at the top and bottom there is no timing difference. The intermediate locations on the left side of the haltere cross-section experience a spiking lag as a consequence of rotation. Conversely, the right side locations experience a leading time difference. This timing difference for a single location is between 0.2 and 1 ms, equal to or larger than the spike timing variability of 0.2 ms between stroke cycles observed in electro-physiological recordings of crane fly halteres [12]. (Online version in colour.)

Spike timing is a function of angular position around the haltere base (figure 5). For the flapping haltere, the spikes for the dorsal side occur simultaneously, and they are in anti-phase with the spikes on the ventral side (vertical black lines). With rotation, the left side of the haltere experiences a delay in spike timing that increases with closer proximity to the lateral margin. Interestingly, the right side experiences a spike timing difference of similar magnitude, yet here it precedes the spikes from flapping alone. Only when rotation is present does spiking occur at the lateral margins, and it does so at double the flapping frequency.

Our predictions of spike timing differences in the presence of Coriolis forces exceed the jitter observed in spike timing of haltere campaniform sensilla (recordings had a mean jitter of approx. 0.2 ms, [12]). This jitter represents the temporal error with which neurons respond to repeated sinusoidal stimuli. Thus, we predict that haltere strains processed through a neural filter are observable by the insect neural system.

## 4. Discussion and conclusion

Using finite-element models, we have shown how fly halteres respond to complex forces and, in turn, how their form influences their function. In contrast to prior studies which modelled the haltere as a point mass on a massless rod [6,8], here we show that complex spatio-temporal patterns of strain emerge from rectilinear and rotational accelerations of the haltere mass. Though previous models were crucial to understanding and interpreting haltere neural encoding and haltere-mediated behaviours, they ignored strains that might occur due to the distributed mass of the haltere and the three-dimensional structure of its base. We show that halteres with distributed mass exhibit twisting deformations in their stalks, and these deformations may be essential to haltere function. Such twisting deformations were shown to contribute to the sensory activity in the campaniform sensilla of moth wings [17], and we show here that similar forces act on the haltere. Halteres evolved from hindwings, and, despite the dramatic alteration of the ancestral wing shape, we demonstrate here that these similar forces also influence sensing capabilities in halteres.

It is important to note that the geometries we used in this study are deliberately simplified in order to understand general behaviours of distributed masses responding to various accelerations. Moreover, we used linear material properties whose values were inspired by prior measurements [21]. Additionally, the magnitudes we report here depend on the frequencies of flapping and the rates of body rotation, which could vary immensely among diverse taxa and conditions. Despite these limitations, we can draw general conclusions about the modes of deformations and their relationship to geometric aspects of halteres.

### 4.1. Coriolis and centrifugal forces deform halteres

Coriolis forces generate torsional moments about the stalk. The resultant twisting depends strongly on stalk geometry, and we demonstrate that a torsionally compliant stalk has over 20 times more twist than the tubular stalk used in other simulations. Haltere bulbs show considerable morphological asymmetry [19], and our model shows that this asymmetry contributes to torsion in the haltere. We also find that if the centre of mass of the haltere is mounted asymmetrically outside of the flapping plane, centrifugal forces cause an out-of-plane bending as well as haltere twisting, regardless of the body’s rotation. This bending may also influence sensing ability of the haltere campaniform sensilla, and has not been previously considered. It would be interesting to explore in more detail the vast range of haltere bulb and stalk morpholpogies in the context of their responses to both Coriolis and centrifugal forces. Additionally, for asymmetric halteres, we found that the spike timing is altered only slightly by the additional centrifugal force associated with a horizontal asymmetry of the bulb (figure 3). Unlike the results shown here, how asymmetry plays into the timing of spikes depends quite strongly on the value of parameters used here and represents an interesting future direction for understanding the sensory consequences of variation in haltere morphology.

### 4.2. Predictions of spike times from strain patterns provide a mechanism for sensing rotation

As the fly’s body rotates in the yaw plane, the timing of the spikes in neurons from campaniform sensilla at various locations on the haltere will change (figure 5). These spike timing changes were predicted 70 years ago by Pringle [6] as a primary mechanism of encoding rotations; however, technological limitations prevented him from observing the spiking activity of single neurons. Recently, single haltere neurons were shown to shift their spike timing when the haltere was oscillated dorso-ventrally (i.e. simulating flapping flight) in various lateral sweep locations (roughly simulating the position of the haltere when it is pushed out of its natural stroke plane by a yaw body rotation) [20]. These experiments provided support for Pringle’s hypothesis, and the results here are in agreement with them.

In the proposed mechanism, a laterally positioned campaniform sensillum would excite a downstream neuron at different times in response to body rotations of different velocities. The timing of the post-synaptic excitation from this sensillum could then be compared with the timing of excitation from a dorsal or ventral sensillum, which do not shift their spike times with body rotation (figure 5). This simple time shift of a single spike would provide a mechanism for a single central neuron to determine if the body is rotating, and its rotation speed could be determined by the elapsed time between post-synaptic excitations. When looking at the timing of action potentials of campaniform sensilla along the circumference of the haltere base, we find that the spike timing changes beyond the jitter threshold [12] as a result of body rotation.

We also note that the maximum phase shifts occurred in campaniform sensilla located on the anterior and posterior aspects (i.e. the lateral part of the stalk cross-section) on our haltere model. Campaniform sensilla located on the dorsal and ventral aspects (i.e. the top and bottom of the model’s stalk) did not shift their phase significantly during rotations, maintaining a consistent firing phase with respect to the haltere’s flapping. This result is consistent with the prediction by Pringle that sensilla of the dorsal scapal plate would maintain the firing time, and the sensilla of the dorsal basal plate would shift their timing during rotations [6].

We note that Pringle based this hypothesis on the orientation of the sensilla themselves: the domes of the dorsal scapal plate are oriented with maximal sensitivity in the plane parallel to the haltere stalk, whereas the domes of the dorsal basal plate are about 30° rotated from this plane. In our simulations, the nodes of the model are not endowed with a specific orientation sensitivity; rather their spike timing is based on the spanwise strain (*ε*_*xx*_) close to the base of the haltere, as it is the dominant strain.

### 4.3. Multiple nonlinearities set limits for detectable rotation rates

While the Coriolis force is linearly proportional to the rotation rate, the nonlinearities of the haltere primary afferents can include a threshold below which they may not fire, resulting in a minimum Coriolis force (and thus a minimum rotation rate) that is necessary for specific primary afferents to fire spikes. The primary afferent neurons synapse onto motoneurons and interneurons that perform their own computations with the input they receive from the haltere. In some models of these neurons [9,20], they act as coincidence detectors, requiring simultaneous input from multiple sensilla before firing. The coincidental firing of multiple afferents is facilitated by the change in spike time that occurs during rotations (figure 5, [20]). At lower rotation rates, the spike timing differences will be smaller (figure 5) and, at some locations along the circumference, the difference might be of the same order as the jitter. This may set a lower limit for detectability, as the ratio of in-plane over out-of-plane bending depends on flapping frequency $f_{\phi }$ (see electronic supplementary material). One possible consequence of this limit is that slower body rotations will not be sensed at higher wingbeat frequencies. In some species, then, it may be advantageous to limit body rotations to short, high-speed bursts like the body saccades seen in *Drosophila* [32]. These saccades are known to be useful for increasing the duration of stable vision, thus minimizing visual motion blur, and allow extraction of spatial information [33–35]. We suggest they may also ensure that the halteres are best able to detect and control the body’s movement. Indeed there are head roll reflexes in response to haltere feedback—most of those occur at high rotation rates, whereas for slower rotation rates head roll responses mostly follow the slower vision-based feedback [33,36].

In this paper, we focus only on yaw perturbation on a single haltere, though saccades can occur with a combination of rotation about all three axes [37]. It is thus possible that the strain at the base of both halteres contains enough information to disentangle rotations about all three axes [8].

### 4.4. Finite-element modelling reveals new sensing modes and mechanisms

Using 3D finite-element models of simplified geometries, combined with neural encoding mechanisms, we show that the shape halteres can strongly influence timing of neuronal activity of mechanosensory cells distributed around the base of halteres. That timing will depend upon the rotation speed and flapping frequency and can be encoded and used to guide steering behaviours in a mechanism that is much faster than in the visual system. Moreover, we have shown that the haltere’s shape plays a profound role in the patterns of haltere deformation. Though halteres evolved in only two insect orders (Diptera and Strepsiptera) [38], the general stalk-and-knob shape has been remarkably conserved, with variations on the theme found in different flies with different stalk lengths, bulb sizes and stalk/bulb ratios [19]. By melding concepts from computational neuroscience, such as stimulus feature selectivity, with three-dimensional structural dynamics of a mechanosensory organ, we gain new insight into the functional organization of halteres. The approach can provide inroads to future work on the vast diversity of haltere geometries found among the Diptera.

## Supplementary Material

Animation of the spikes along haltere circumference

## Supplementary Material

Animation of haltere simulation kinematics 1

## Supplementary Material

Animation of haltere simulation kinematics 2

## Data accessibility

The simulation data from the computational models are available at Zenodo (https://doi.org/10.5281/zenodo.2542944). We used Matlab [31] to analyse the data. Our code to analyse the finite-element results and to implement the neural encoding can be found at https://github.com/tlmohren/Haltere-code.
