# Supplementary figures and images for: Coriolis and centrifugal forces drive haltere deformations and influence spike timing
Source: J R Soc Interface. 2019 Apr 24;16(153):20190035. doi: 10.1098/rsif.2019.0035 (PMC6505556; doi:10.1098/rsif.2019.0035)

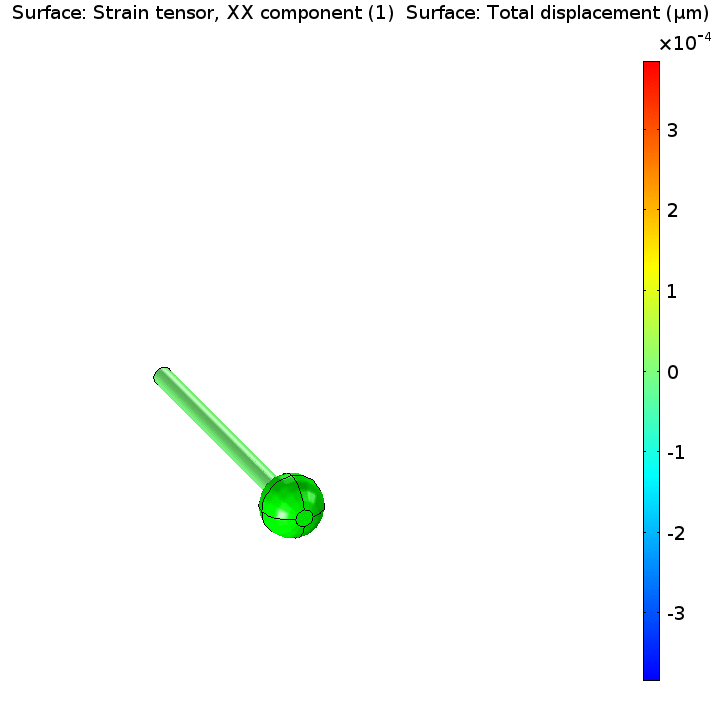

Supplement: Animation of haltere simulation kinematics 1 [file rsif20190035supp2.gif]

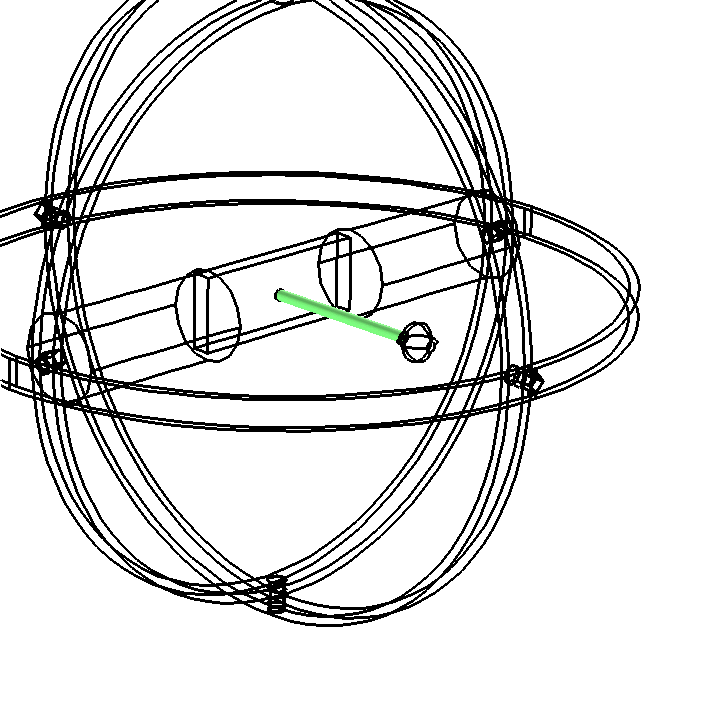

Supplement: Animation of haltere simulation kinematics 2 [file rsif20190035supp3.gif]
